# Programming for increased expression of hippocampal GAD67 mediated the hypersensitivity of the hypothalamic–pituitary–adrenal axis in male offspring rats with prenatal ethanol exposure

**DOI:** 10.1038/s41419-018-0663-1

**Published:** 2018-05-31

**Authors:** Juan Lu, Zhexiao Jiao, Ying Yu, Chong Zhang, Xia He, Qiang Li, Dan Xu, Hui Wang

**Affiliations:** 1 Department of Pharmacology, Basic Medical School of Wuhan University, Wuhan, Hubei Province China; 2 Hubei Provincial Key Laboratory of Developmentally Originated Disease, Wuhan, Hubei Province China; 3 Department of Neurology, Renmin Hospital of Wuhan University, Wuhan, 430060 China; 4 Gansu provincial hospital of TCM Affiliated to Gansu University of Chinese Medicine, Gansu, 730050 China

## Abstract

An imbalance of excitatory and inhibitory signals in the brain has been proposed to be one of the main pathological features of various diseases related to hypothalamic–pituitary–adrenal axis (HPAA) dysfunction. Excessive glutamate release induces neuronal excitotoxicity, while glutamic acid decarboxylase (GAD) 67 promotes the transformation of excessive glutamate to γ-aminobutyric acid (GABA). Our previous studies demonstrated that prenatal ethanol exposure (PEE) causes foetal over-exposure to maternal corticosterone and hypersensitivity of the HPAA after birth, but its intrauterine programming mechanism is unknown. In this study, PEE was shown to lead to an enhanced potential excitatory ability of the hypothalamus and hypersensitivity of the HPAA, as well as mild abnormal hippocampal morphology, demethylation of the -1019 to -691-bp region in the hippocampal GAD67 promoter and upregulation of GAD67 expression accompanied by a reduction in glutamatergic neurons and increase in GABAergic neurons in PEE male offspring. Similar changes were also found in PEE male foetal rats. Furthermore, corticosterone increased the expression of the glucocorticoid receptor (GR) and GAD67 in foetal hippocampal H19-7 cells in a concentration-dependent manner, accompanied by demethylation of the GAD67 promoter, a decrease in glutamatergic neurons and increase in GABAergic neurons. The GR inhibitor, mifepristone, reversed the effects of corticosterone on H19-7 cells. These results suggested that PEE-induced excessive corticosterone can lead to upregulation of GAD67 through epigenetic modification mediated by the GR in the male foetal hippocampus, thereby weakening the negative regulation of the HPAA by the hippocampus and increasing the potential excitatory ability of the hypothalamus. These changes persisted until after birth, resulting in hypersensitivity of the HPAA. However, gender differences were observed in the hippocampal development, morphology and GAD67 expression associated with PEE. Programming for the increased expression of hippocampal GAD67 is a potential mechanism responsible for the hypersensitivity of the HPAA in PEE male rats.

## Introduction

The hypothalamic–pituitary–adrenal axis (HPAA) is an important neuroendocrine axis involved in the stress response and metabolic regulation. A large number of studies have suggested that an adverse intrauterine environment can cause developmental programming alterations of the HPAA and have a permanent effect on neuroendocrine function^1–3^. The pathogenesis of abnormal developmental HPAA programming is the final common pathway in foetal-originated metabolic syndrome and a series of emotional disorders^4–9^. Our previous research introduced a mechanism of “HPAA-associated neuroendocrine metabolic programming alteration” to explain the increased susceptibility to metabolic diseases of intrauterine growth retardation (IUGR) offspring rats with prenatal ethanol exposure (PEE)^10^. The mechanism may be associated with over-exposure of the foetus to elevated maternal glucocorticoids resulting from impaired placental glucocorticoid barriers. Excessive maternal glucocorticoids not only inhibit the development of foetal HPAA function but also alter glucose and lipid metabolism in peripheral tissues, eventually resulting in IUGR^10, 11^. These intrauterine neuroendocrine and metabolic changes can be extended to after birth even into adulthood, embodied by hypersensitivity of the HPAA to chronic stress (CS) and glucocorticoid-dependent changes in glucose and lipid metabolism in peripheral tissues in PEE offspring with a post-weaning high-fat diet, thereby causing metabolic syndrome and non-alcoholic fatty liver disease^10, 12^. However, whether hypersensitivity of the HPAA to CS also exists in PEE offspring fed a normal diet, whether gender differences exist in the mechanism resulting in HPAA hypersensitivity and whether epigenetic modification is involved in the hypersensitivity of the HPAA are unclear.

The hypothalamic paraventricular nucleus (PVN) directly controls the activity of the HPAA. During stress, corticotrophin-releasing hormone (CRH) and arginine vasopressin (AVP) are secreted from parvocellular neurons in the PVN to stimulate the secretion of adrenocorticotropic hormone (ACTH) from the pituitary gland. ACTH subsequently promotes the release of glucocorticoids (corticosterone in rodents) from the adrenal cortex. The hippocampus, as the advanced negative control centre of the HPAA, not only suppresses the stress response of the HPAA but also restores the excessive stress state of the HPAA to baseline levels through governing the negative regulation of the hypothalamus^13^. Glutamate and γ-aminobutyric acid (GABA) are, respectively, important excitatory and inhibitory neurotransmitters in the mammalian brain, and the dynamic balance between them maintains the activities of multiple brain regions, including the hippocampus^14^. Glutamic acid decarboxylase (GAD) is the rate-limiting enzyme in the synthesis of GABA by decarboxylation of glutamate into GABA, playing a vital role in the glutamate and GABA balance.

In this study, we observed PEE-induced dysfunction of the HPAA in male IUGR offspring rats before and after birth, as well as in adulthood with CS. Additionally, we explored the intrauterine programming mechanism resulting in HPAA hypersensitivity in PEE offspring rats by examining hypothalamic excitatory/inhibitory neuronal differentiation and hippocampal negative regulation dysfunction. This study provides important theoretical value and practical significance for clarifying the hippocampal neurotoxic mechanism of ethanol/alcohol, understanding the intrauterine programming of adult diseases associated with HPAA dysfunction, and improving the quality of life of the population.

## Results

### Adult offspring rats

#### Birthweight, HPAA activity and potential hypothalamic excitatory ability

**Fig. 1:**
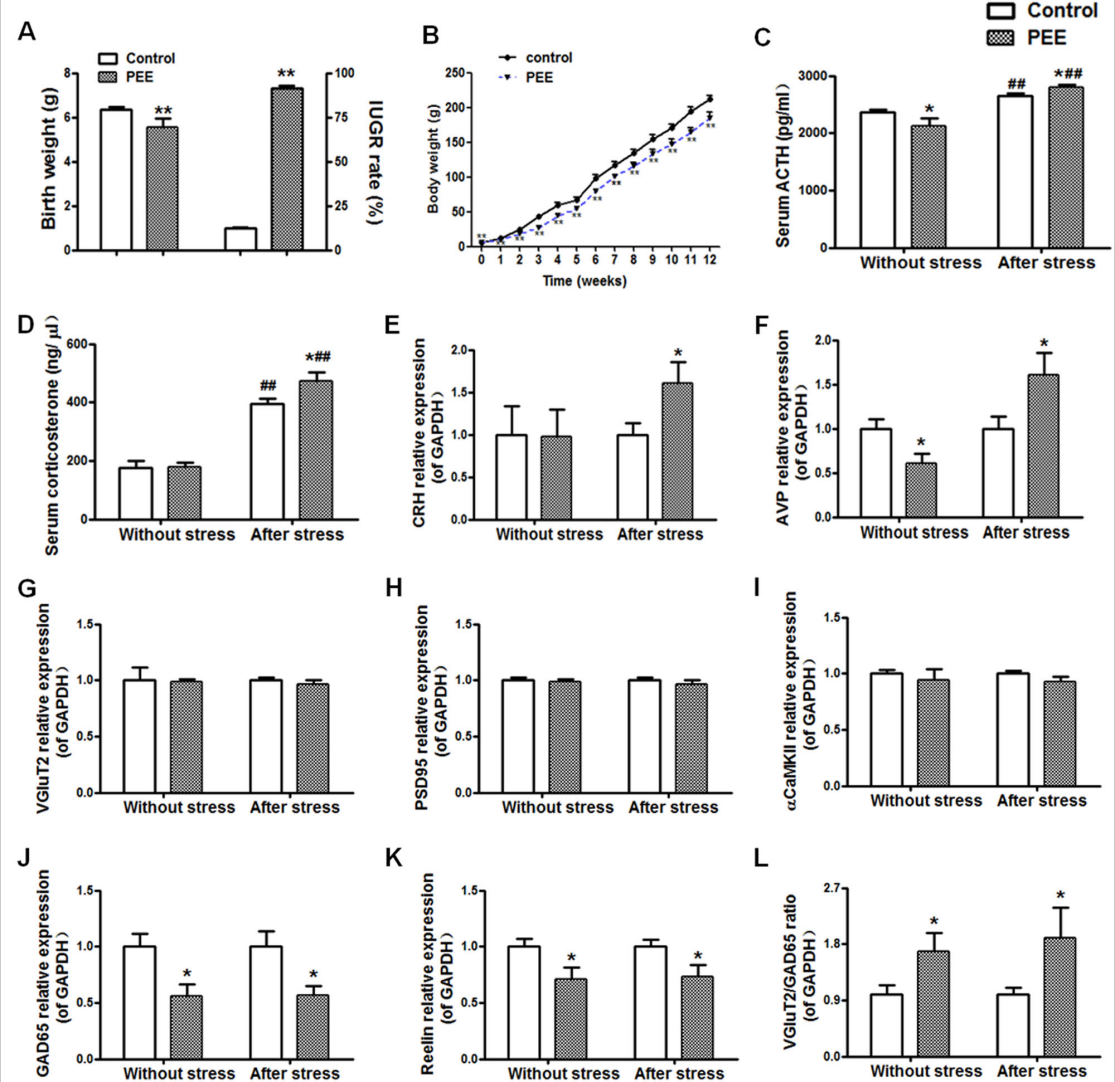
Effects of prenatal ethanol exposure (PEE, 4  g/kg per day) on the bodyweight, intrauterine growth retardation (IUGR) rate, hypothalamic–pituitary–adrenal axis activity and potential hypothalamic excitatory ability without and after chronic stress in male adult offspring rats. **a** Birthweight and IUGR rate. **b** Bodyweight after birth. **c**, **d** Serum adrenocorticotropic hormone (ACTH) and corticosterone concentrations. **e**–**k** Hypothalamic corticotrophin-releasing hormone (CRH), arginine vasopressin (AVP), vesicular glutamate transporter 2 (VGluT2), post-synaptic density-95 (PSD95), Ca^2+^/calmodulin-dependent protein kinase II-α (α-CaMKII), glutamic acid decarboxylase 65 (GAD65) and Reelin mRNA expression. **l** Hypothalamic expression ratio of VGluT2/GAD65. Mean ± S.E.M., *n* = 8 offspring from eight litters. ^*^P < 0.05, ^**^P < 0.01 vs. control; ^#^*P* < 0.05, ^##^*P* < 0.01 vs. without stress

Consistent with the results of our previous studies^12, 15^, our results confirmed that PEE can cause low birthweight and high IUGR rate in male offspring rats (Fig. 1a, b). Moreover, using this stable IUGR rat model induced by PEE, we demonstrated a hypersensitivity of the HPAA to CS in male adult offspring, embodied by increased expression levels of CRH and AVP in the hypothalamus and elevated levels of serum ACTH and corticosterone after CS (Fig. 1c–f). We next observed the potential excitatory activity of the hypothalamus, which is the direct control centre of the HPAA. GAD65 is the major synthase of GABA, and Reelin is involved in its synaptic transmission and plasticity. Vesicular glutamate transporter 2 (VGluT2) is a key transporter of glutamate, while post-synaptic density 95 (PSD95) and Ca^2+^/calmodulin-dependent protein kinase II-α (α-CaMKII) are marker proteins of glutamatergic neurons. VGluT2 and GAD65 are considered to be specific markers of glutamatergic and GABAergic nerve fibres^16, 17^. Results showed significant downregulation of hypothalamic GAD65 and Reelin expression in PEE male offspring both without and after CS, accompanied by an increase in the VGluT2/GAD65 expression ratio with no significant changes in VGluT2, PSD95 or α-CaMKII (Fig. 1g–l). These findings suggested that PEE may enhance the potential excitatory ability of the hypothalamus in male offspring rats mainly through downregulation of the expression of GABAergic neuronal proteins in the hypothalamus both without and after CS.

#### Hippocampal morphology, the IGF1 signalling pathway, and the glutamatergic and GABAergic neuron balance

**Fig. 2:**
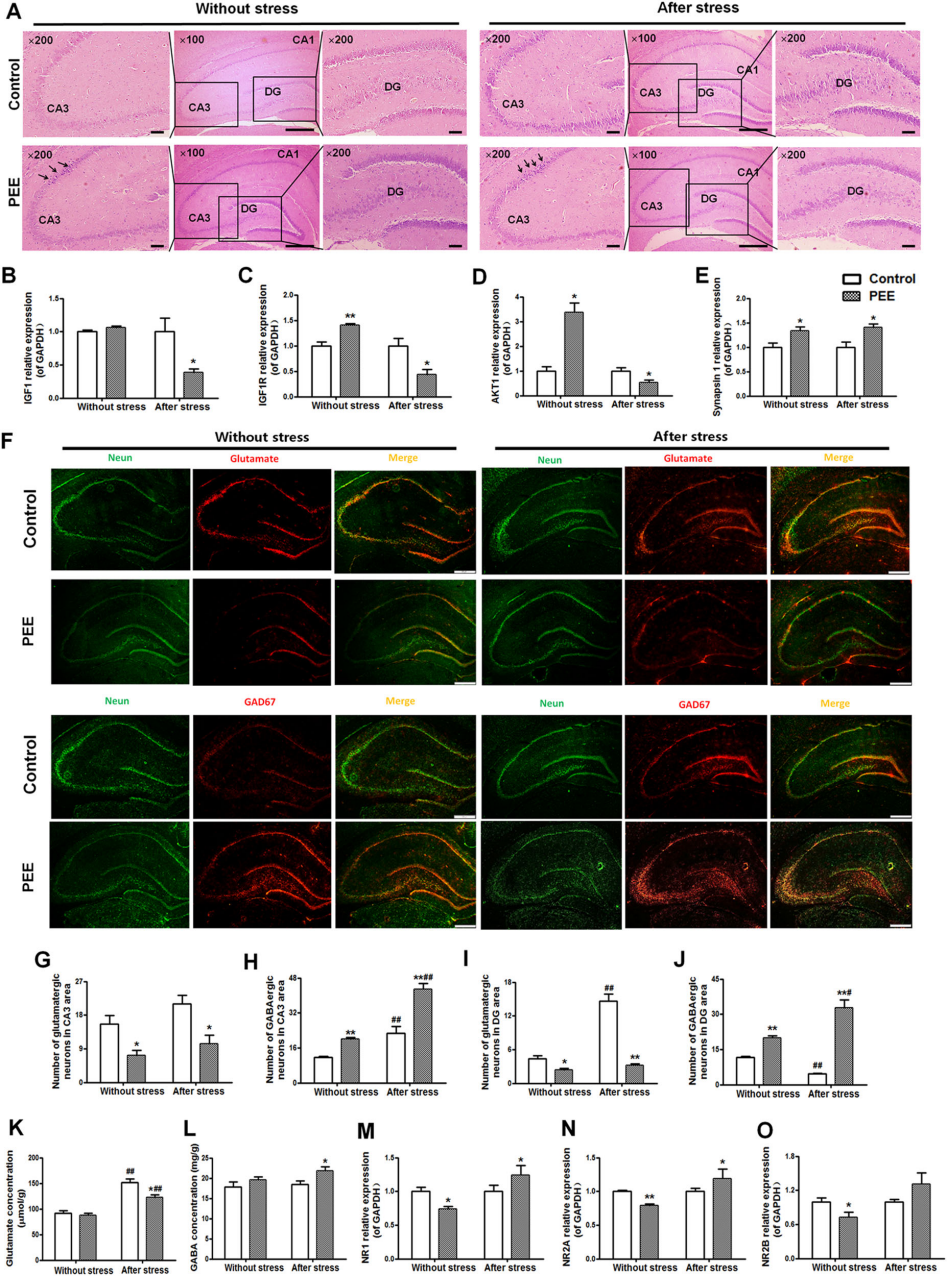
Effects of prenatal ethanol exposure (PEE, 4 g/kg per day) on hippocampal morphology, glutamatergic and GABAergic neuron numbers, and mRNA expression of insulin-like growth factor 1 (IGF1) signalling pathway-related genes and *N*-methyl-D-aspartate-subtype glutamate receptors (NRs) without and after chronic stress in male adult offspring rats. **a** Morphologic changes in the whole hippocampus (HE, × 100), granular cells in the dentate gyrus (DG) areas and pyramidal cells in the cornu ammonis 3 (CA3) (HE, × 200). There were only a few neuronal nuclei in the CA3 region that were dense and darkly stained in the PEE group both without and after CS. **b**–**e** Hippocampal IGF1, IGF1 receptor (IGF1R), protein kinase B (AKT1) and synapsin 1 mRNA expression. **f** Photomicrographs of immunofluorescence-labelled glutamatergic neurons (glutamate, red), GABAergic neurons (GAD67, red) and neurons (NeuN, green). Scale bars = 200 μm. **g**–**j** Quantitative analysis of the glutamatergic or GABAergic neurons in the hippocampal CA3 and DG areas; three brain sections from the same levels of the hippocampus were selected from each animal and quantified. **k**, **l** Hippocampal glutamate and gamma aminobutyric acid (GABA) concentrations. **m**–**o** Hippocampal NR1, NR2A, and NR2B expression. Mean ± S.E.M., *n* = 8 offspring from eight litters. ^*^P < 0.05, ^**^P < 0.01 vs. control; ^#^*P* < 0.05, ^##^*P* < 0.01 vs. without stress

We further investigated functional and morphological changes of the hippocampus, which is the advanced regulative centre of the HPAA. HE staining showed that there were only a few neuronal nuclei in the cornu ammonis (CA) three region that were dense and darkly stained in the PEE group both without and after CS (Fig. 2a). The insulin-like growth factor 1 (IGF1) signalling pathway (and the related genes including IGF1, IGF1 receptor (IGF1R) and protein kinase B (AKT1)) is known to be closely related to hippocampal neuronal development and synaptogenesis^18^, and synapsin 1, as a stress response gene, regulates the release of hippocampal neurotransmitters^19^. The findings indicated that PEE can induce morphological damage to hippocampal tissue (especially in the CA3 area) and upregulate the IGF1 signalling pathway (higher expression levels of IGF1R and AKT1) and synapsin 1 expression (Fig. 2b–e), whereas the IGF1 signalling pathway was downregulated after CS.

We also quantified the number of hippocampal glutamatergic and GABAergic neurons, the neurotransmitter concentrations and the expression levels of neurotransmitter receptors. In the immunofluorescence analysis, glutamate and GAD67 were used to represent the glutamatergic and GABAergic neurons, respectively (Fig. 2f). The quantitative analysis showed that compared to the number of glutamatergic neurons in the control group, there were significantly fewer glutamatergic neurons in the hippocampal CA3 and granular cells in the dentate gyrus (DG) areas of the PEE group (Fig. 2g, i) and significantly more GABAergic neurons in the PEE groups both without CS and after CS (Fig. 2h, J). The hippocampal glutamate and GABA concentrations in the PEE group without CS were no different than the concentrations in the control group, but the glutamate concentration was significantly lower, while the GABA concentration was significantly higher in the PEE group after CS compared to the concentrations in the control groups (Fig. 2k, l). The expression levels of hippocampal glutamate receptors, *N*-methyl-d-aspartate-subtype glutamate receptor (NR) 1, NR2A and NR2B, in the PEE group were downregulated without CS but upregulated after CS (Fig. 2m–o) when compared with the corresponding control levels.

#### Hippocampal GR and GAD67 expression and epigenetic modifications of GAD67

**Fig. 3:**
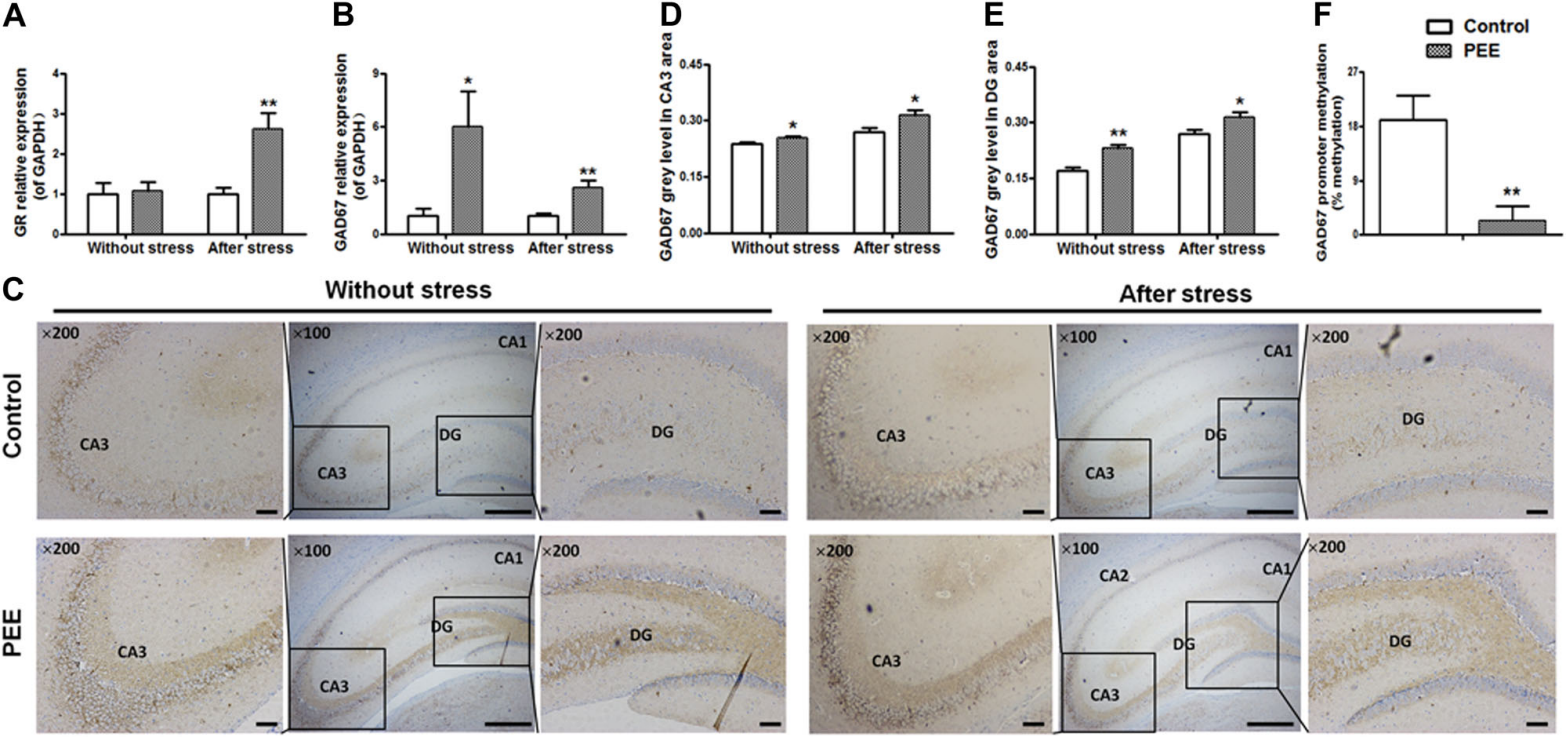
Effects of prenatal ethanol exposure (PEE, 4  g/kg per day) on hippocampal glucocorticoid receptor (GR) and glutamic acid decarboxylase 67 (GAD67) expression and the total methylation rate of the GAD67 promoter (**-**1019 to -691 bp) without and after chronic stress in male adult offspring rats. **a**, **b** Hippocampal GR and GAD67 mRNA expression. **c** Photomicrographs of immunohistochemistry for hippocampal GAD67; the brown signal was distributed in the cytoplasm and vesicles of hippocampal neurons. **d**, **e** Quantitative analysis of GAD67 in the hippocampal CA3 and DG areas; three brain sections from different levels of the hippocampus were selected from each animal and quantified. **f** Total methylation rate of the GAD67 promoter (-1019 to -691 bp); % methylation was used to quantify the methylations status of the GAD67 promoter (-1019 to -691 bp). Mean ± S.E.M., *n* = 8 offspring from eight litters. ^*^P < 0.05, ^**^P < 0.01 vs. control

The glucocorticoid receptor (GR) is known to mediate the negative regulation of the HPAA by the hippocampus, and GAD67 is a regulatory enzyme that balances the excitatory/inhibitory signal. We further investigated the expression levels of hippocampal GR and GAD67 in adult male rats with PEE. The results showed that the expression level of hippocampal GR was unchanged without CS but significantly increased after CS in the PEE male rats compared with the levels in the control group (Fig. 3a). Meanwhile, the expression levels of hippocampal GAD67 were upregulated significantly both without and after CS in the PEE group compared to the levels in the control group (Fig. 3b). The immunohistochemistry staining showed that the brown GAD67 signal was distributed in the cytoplasm and vesicles of hippocampal neurons (Fig. 3c). Compared with the levels in the control group, the protein expression levels of GAD67 in the hippocampal DG and CA3 areas were significantly upregulated in the PEE group both without and after CS (Fig. 3d, e). In addition, the bisulfite sequencing PCR (BSP) results showed that the total methylation rate in the -1019 to -691-bp region of the hippocampal GAD67 promoter was significantly lower in the PEE male offspring rats than in the control group without CS (Fig. 3f). These observations might suggest that PEE induced demethylation of the GAD67 promoter and increased the expression of hippocampal GAD67. Moreover, the expression of GR was significantly higher and the expression of GAD67 remained higher in the PEE male rats than in the control rats exposed to CS.

### Foetal rats

#### Serum corticosterone concentration and potential hypothalamic excitatory ability

**Fig. 4:**
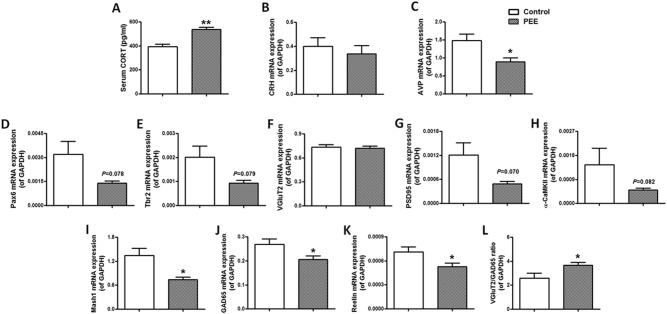
Effects of prenatal ethanol exposure (PEE, 4  g/kg per day) on the serum corticosterone (CORT) concentration and potential hypothalamic excitatory ability in male foetal rats. **a** Serum CORT concentration. **b**–**k** Hypothalamic expression of corticotrophin-releasing hormone (CRH), arginine vasopressin (AVP), paired box 6 (Pax6), T-box brain protein 2 (Tbr2), vesicular glutamate transporter 2 (VGluT2), post-synaptic density 95 (PSD95), Ca^2+^/calmodulin-dependent protein kinase II-α (α-CaMKII), mammalian achaete-scute homologue-1 (Mash1), glutamic acid decarboxylase 65 (GAD65) and Reelin. **l** Hypothalamic expression ratio of VGluT2/GAD65. Mean ± S.E.M., *n* = 8 litters. ^*^P < 0.05, ^*^P < 0.01 vs. control

To investigate whether the enhanced potential excitability of the hypothalamus in PEE male offspring originated intrauterine, we analysed the hypothalamic-related indexes in the PEE male foetuses. As paired box 6 (Pax6), T-box brain protein 2 (Tbr2) and mammalian achaete-scute homologue-1 (Mash1) are important transcriptional factors that govern glutamatergic/GABAergic differentiation during foetal neuronal development^20–22^, transient expression of intrauterine Pax6, Tbr2 and Mash1 can induce persistent changes in the expression of glutamatergic proteins including VGluT2, PSD95 and α-CaMKII, and GABAergic proteins including GAD65 and Reelin^22^. We further found that there was an increase in the serum corticosterone level (Fig. 4a), a trend towards a reduction in hypothalamic Pax6, Tbr2, PSD95 and α-CaMKII expression, and a significant downregulation in Mash1, Reelin and GAD65 expression but an increase in the expression ratio of VGluT2/GAD65 in the foetuses (Fig. 4b–l). These results suggested that the PEE-induced high-glucocorticoid level in foetal rats may inhibit GABAergic differentiation and upregulate the equilibrium point between excitability and inhibitory signals of the foetal hypothalamus, resulting in an enhanced potential excitatory ability of the foetal hypothalamus, thereby mediating the initial hypersensitive response of the HPA axis to CS.

#### Hippocampal morphology, the IGF1 signalling pathway and the glutamatergic and GABAergic neuron balance

**Fig. 5:**
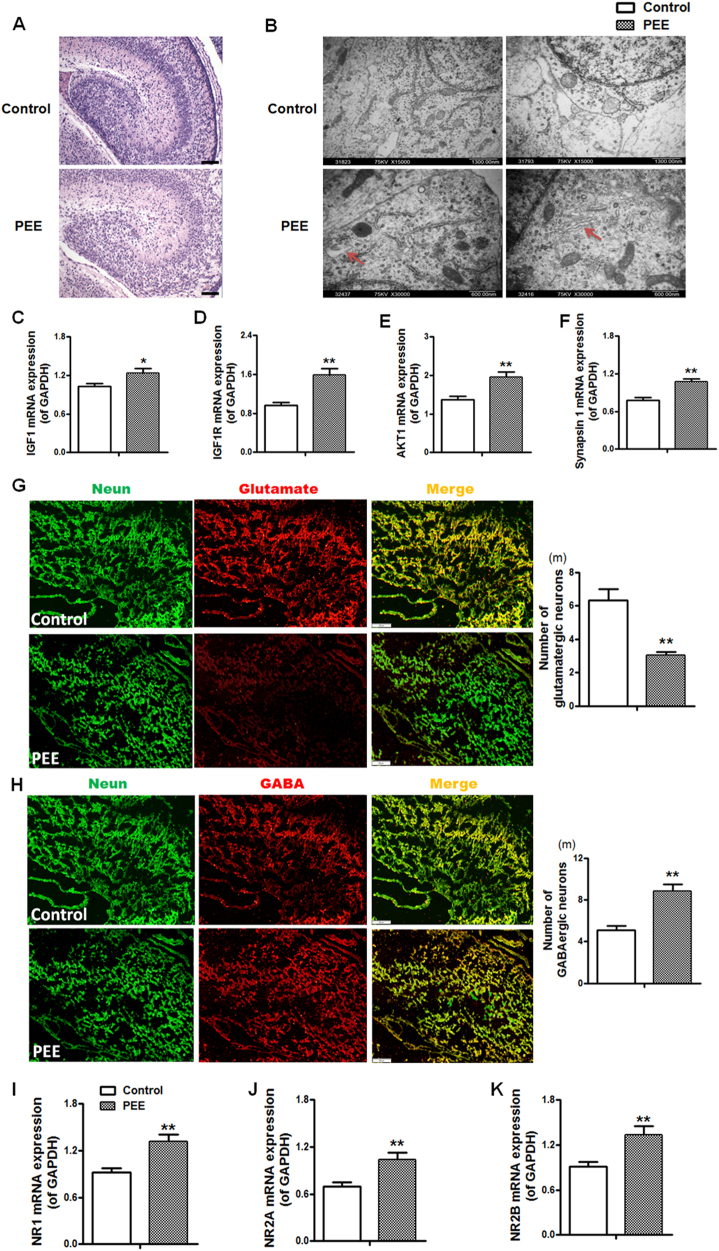
Effects of prenatal ethanol exposure (PEE, 4  g/kg per day) on hippocampal morphology, the number of glutamatergic and GABAergic neurons, and expression of insulin-like growth factor 1 (IGF1) signal pathway-related genes and *N*-methyl-D-aspartate-subtype glutamate receptors (NRs) in male foetal rats. **a** The morphology of the foetal hippocampus (HE, ×400). **b** The ultrastructure of the foetal hippocampus (Transmission electron microscopy, ×8000, ×15,000). Visible dilatation of the endoplasmic reticulum and hypertrophy of the golgi body are shown in the foetal hippocampal neurons of the PEE group. **c**–**f** Hippocampal expression of IGF1, IGF1 receptor (IGF1R), protein kinase B (AKT1) and synapsin 1. **g**, **h** Photomicrographs and quantitative analysis of immunofluorescence for glutamatergic neurons (glutamate, red), GABAergic neurons (GAD67, red) and neurons (NeuN, green). Scale bars = 200 μm. Quantitative analysis of glutamatergic and GABAergic neurons in the whole hippocampus; three brain sections from different levels of the hippocampus were selected from each animal and quantified. **i**–**k** Hippocampal expression of NR1, NR2A and NR2B. Mean ± S.E.M., *n* = 8 litters. ^*^P < 0.05, ^**^P < 0.01 vs. control

Furthermore, we investigated the hippocampal morphology, expression of IGF1 signalling pathway-related genes and synapsin 1 in the male foetuses with PEE. The results of the HE staining showed no obvious morphological difference between the control group and the PEE group (Fig. 5a). Further observations of the foetal hippocampal ultrastructure under electron microscopy showed visible dilatation of the endoplasmic reticulum and hypertrophy of the golgi body in the foetal hippocampal neurons of the PEE group (Fig. 5b). The expression levels of IGF1 signalling pathway-related proteins and synapsin 1 were increased in the PEE male foetal hippocampus compared to the levels in the control group (Fig. 5c–f). These results indicated that the ultrastructure of the male foetal hippocampus was slightly affected by PEE and that the IGF1 signalling pathway and synapsin 1 expression levels were enhanced by PEE.

We further quantified the glutamatergic and GABAergic neurons and the expression levels of glutamate receptors in the foetal hippocampus. Compared with the number of glutamatergic and GABAergic neurons in the control group, quantitative analysis of immunofluorescence showed significantly fewer glutamatergic neurons (Fig. 5g) and significantly more GABAergic neurons (Fig. 5h) in the PEE group, and the quantitative real-time RT-PCR (qRT-PCR) results showed significantly higher expression of NR1, NR2A and NR2B in the male foetal hippocampus with PEE than in the control hippocampus (Fig. 5i–k).

#### Hippocampal glucocorticoid metabolic activation system, GAD67 expression and epigenetic modifications

**Fig. 6:**
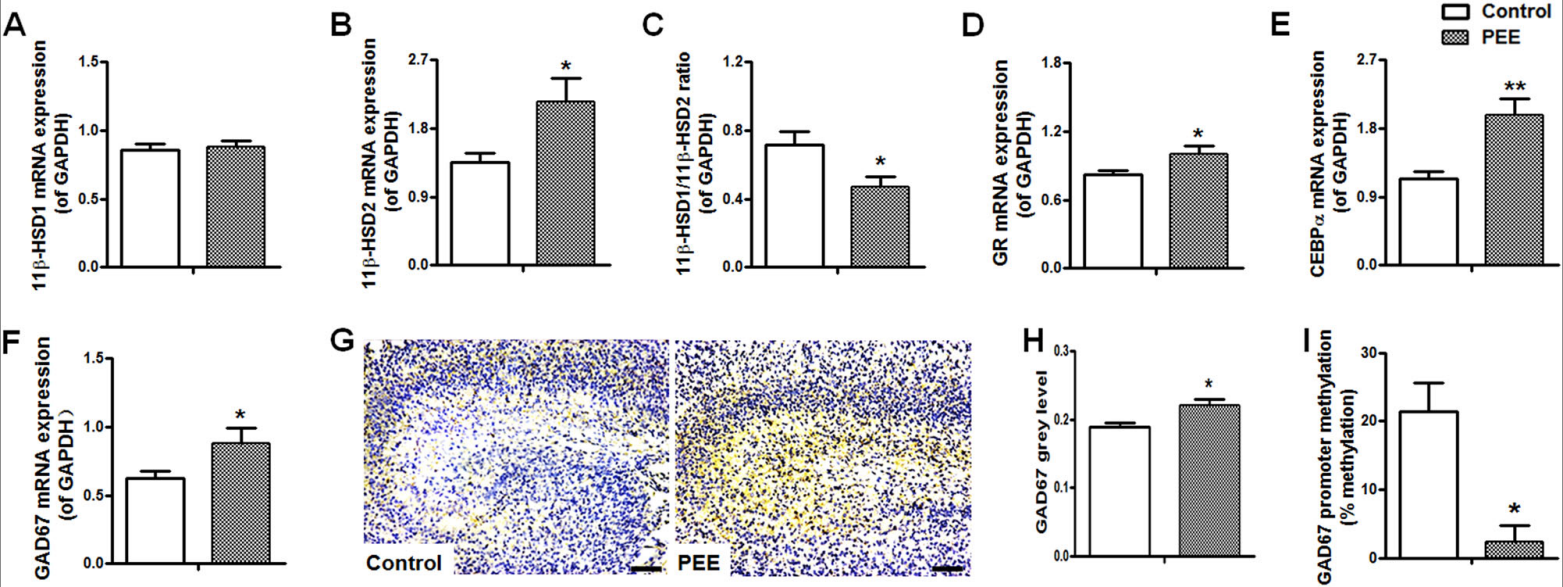
Effects of prenatal ethanol exposure (PEE, 4  g/kg per day) on the expression of hippocampal glucocorticoid metabolic activation system and glutamic acid decarboxylase 67 (GAD67) expression and the total methylation rate of the GAD67 promoter region (-1019 to -691 bp) in male foetal rats. **a**–**f** The mRNA expression of the metabolic activation system (11β-hydroxysteroid dehydrogenases (11β-HSDs), 11β-HSD1/11β-HSD2 expression ratio, glucocorticoid receptor (GR), CCAAT enhancer-binding protein α (C/EBPα) and GAD67. **g** Photomicrographs of immunohistochemistry for hippocampal GAD67; the brown signal was distributed in the whole hippocampus. **h** Quantitative analysis of GAD67 in the hippocampus; three brain sections from different levels of the hippocampus were selected from each animal and were quantified. **i** Total methylation rate of the GAD67 promoter region (-1019 to -691 bp); % methylation was used to quantify the methylations status of the GAD67 promoter (-1019 to -691 bp). Mean ± S.E.M., *n* = 8 litters. *P < 0.05, **P < 0.01 vs. control

Moreover, we quantified the mRNA expression levels of foetal hippocampal glucocorticoid metabolic activation system and GAD67, as well as the methylation status of the GAD67 promotor region. Compared with the expression levels in the control group, the expression levels of 11β-hydroxysteroid dehydrogenases (11β-HSD) 2, GR, CCAAT enhancer-binding protein (C/EBP) α and GAD67 in the hippocampus were significantly upregulated, and the 11β-HSD1/11β-HSD2 expression ratio was downregulated in the PEE male rats (Fig. 6b–f). Immunohistochemistry staining showed that the brown signal for GAD67 was widely distributed in the foetal hippocampus (Fig. 6g). Compared with the expression in the control group, the protein expression level of GAD67 in the hippocampus was significantly upregulated in the PEE group (Fig. 6h). The BSP results also showed that the rate of total methylation in the -1019 to -691-bp region of the GAD67 promoter in the foetal hippocampus with PEE was much lower than in the control hippocampus (Fig. 6i). Therefore, glucocorticoid metabolic activation and inactivation co-existed in the male foetal hippocampus with PEE. Additionally, these results suggested that PEE can induce demethylation in the GAD67 promoter and increase GAD67 expression in the male foetal hippocampus.

### Foetal hippocampal cell line

#### Glutamatergic/GABAergic neuron numbers and the expression and epigenetic modifications of GAD67

**Fig. 7:**
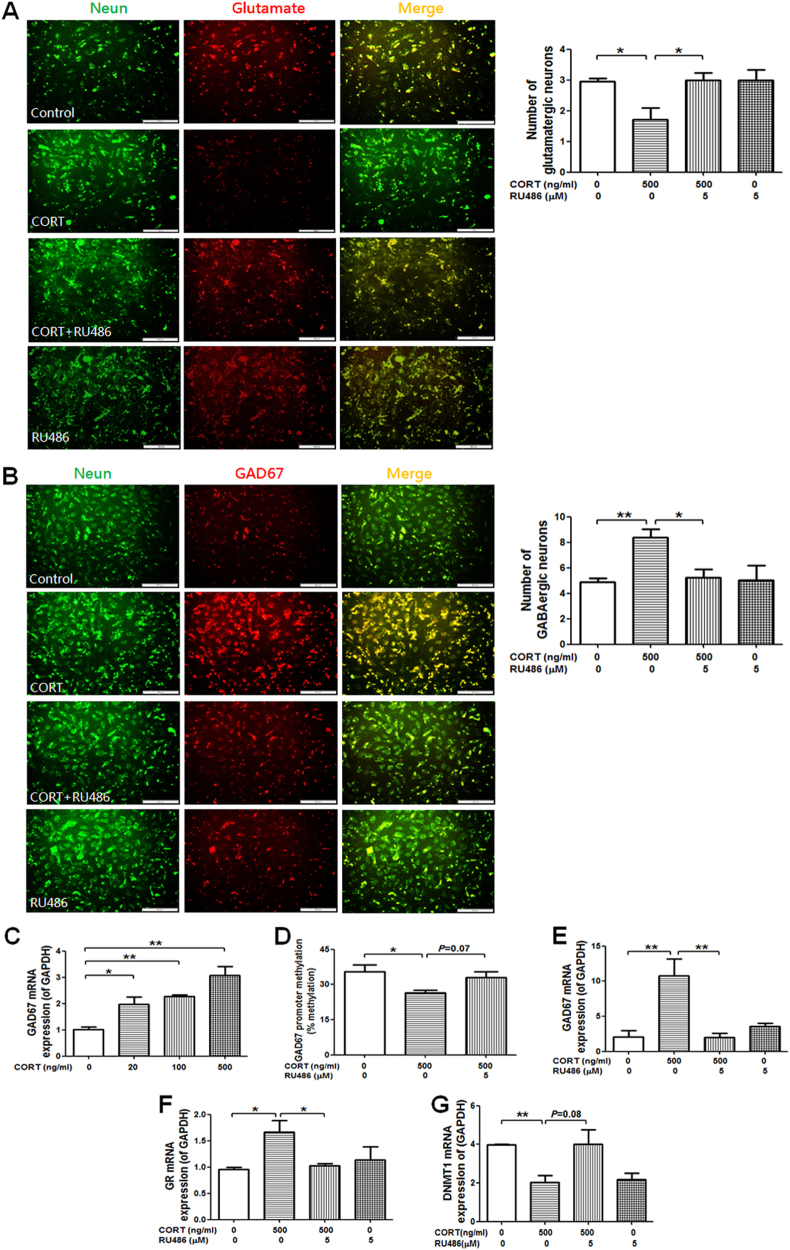
Effects of corticosterone (20–500  ng/ml) and mifepristone (RU486, 5  μM) on glutamatergic (glutamate)/GABAergic (GAD67) neuron numbers, the expression of glucocorticoid receptor (GR), glutamic acid decarboxylase 67 (GAD67) and DNA methylation transferase 1 (DNMT1) and the total methylation rate of the GAD67 promoter region (-1019 to -691 bp) in the foetal hippocampal cell line H19-7. **a**, **b** Photomicrographs and quantitative analysis of immunofluorescence for glutamatergic neurons (glutamate, red), GABAergic neurons (GAD67, red) and neurons (NeuN, green) (Scale bars = 200 μm, *n* = 6). Quantitative analysis of glutamatergic and GABAergic neurons. **c**, **e**–**g** The mRNA expression of GAD67, GR and DNMT1 (*n* = 3). D Total methylation rate of the GAD67 promoter region (-1019 to -691 bp); % methylation was used to quantify the methylations status of the GAD67 promoter (-1019 to -691 bp). Mean ± S.E.M., *n* = 3. ^*^P < 0.05, ^**^P < 0.01 vs. control

According to the above serum corticosterone levels of the PEE foetus, we set the maximum concentration of corticosterone at 500 ng/ml for the in vitro study. To confirm the effect of GR and the high level of corticosterone on foetal hippocampal neuron numbers, cells were treated with 500 ng/ml corticosterone with or without the GR inhibitor mifepristone (RU486, 5 μM) for 5 days. The results indicated that a high level of corticosterone can reduce the number of glutamatergic neurons (Fig. 7a) and increase the number of GABAergic neurons (Fig. 7b). We further observed that the expression level of GAD67 was upregulated in a concentration-dependent manner after treatment with corticosterone (20, 100 and 500 ng/ml) (Fig. 7c). Furthermore, corticosterone (500 ng/ml) alone induced demethylation in the -1019 to -691-bp region of the GAD67 promoter (Fig. 7d), whereas co-treatment with RU486 reversed the above changes. Meanwhile, GR and GAD67 expression was upregulated, while DNA methyltransferase 1 (DNMT1) expression was downregulated by corticosterone (500 ng/ml) alone (Fig. 7e–g). However, the above changes were reversed after co-treatment with RU486 (Fig. 7a, b, d–g), suggesting that these effects were mediated by GR activation.

## Discussion

An increasing number of studies have reported that ethanol has a direct and damaging effect on the hippocampus^23–25^, which can impair the balance of glutamatergic/GABAergic synapses of the hippocampus and further lead to the excitement of pyramidal neurons^26^. Ethanol can be metabolised into acetaldehyde by alcohol dehydrogenase (ADH) 3^27^. As ADH3 is mainly expressed in pyramidal neurons, acetaldehyde generated by high doses of ethanol is most likely to damage pyramidal neurons^28^. Our previous studies confirmed that PEE (4 g/kg per day) resulted in a high ethanol level in foetal serum^10^. In the present study, PEE induced mild changes in the ultrastructure of the hippocampus in male foetal rats. HE staining showed that there were a small number of injured pyramidal neurons in the hippocampal CA3 area of PEE male adult rats both exposed and not exposed to CS. We hypothesise that these changes may be related to the local excitatory injury of the hippocampus caused by the increased release of glutamate from the hippocampus induced by ethanol.

When the glucocorticoid level rises (e.g., stress response), the excess glucocorticoids activate GRs in the hippocampus to release glutamate and further inhibits the CRH neurons in the PVN region, thereby playing a negative regulatory role to avoid excessive activation of the HPAA^29^. GAD plays an important role in the balance of hippocampal excitatory and inhibitory amino acids. Studies have shown that the foetal hippocampus primarily expresses GAD67^30, 31^, while GR has been reported to be co-localised in GAD67-positive neurons^31^. Our previous studies confirmed that PEE can cause over-exposure of the foetus to maternal glucocorticoids^10, 11^. We further found that the expression of GAD67 in the male foetal hippocampus were upregulated and that the number of glutamatergic neurons and the amount of glutamate were decreased by PEE. However, the number of GABAergic neurons and the GABA content were increased. Meanwhile, we confirmed that the glucocorticoid-induced upregulation of GAD67 expression in the cell experiments was mediated by hippocampal GR. Therefore, we hypothesise that the upregulation of GAD67 expression in the hippocampus is a protective compensatory response to reduce the excitotoxicity of excessive glutamate locally in hippocampal tissue through promoting the bioconversion of glutamate to GABA. However, this compensatory effect of the hippocampus attenuated the GABAergic signal, as well as permanently changing the stress onset and sensitivity of the hypothalamus. Interestingly, the upregulation of hippocampal GAD67 was not observed in the PEE female offspring in the same experiment (Supplementary Fig. 1), suggesting that the mechanism for the enhanced potential excitatory ability of hypothalamus is gender-specific^32^.

Correct synaptic formation is the basis of the structure and function of the nervous system and is regulated by a variety of nerve growth factors, including IGF1. As a phosphate protein for nerve cells, synapsin 1 plays a vital role in the release of neurotransmitters. Glucocorticoids have been reported to be able to upregulate the expression of synapsin 1 in the DG/CA3^19^. In the present study, we found that the expression levels of the hippocampal IGF1 signalling pathway and synapsin 1 before and after birth were increased, which persisted into adulthood. We speculate that over-expression of the IGF1 signalling pathway and synapsin 1 in the PEE male foetal hippocampus may be similar to the above increase in GAD67 expression in its role as one of the intrauterine compensatory responses. Interestingly, downregulation and downward trends in the IGF1 signalling pathway (Supplementary Fig. 2) as well as relatively heavy damage (Supplementary Fig. 3) to the female hippocampus were induced by PEE. A large number of studies have suggested that over-exposure to glucocorticoids during pregnancy can affect foetal development^33–35^ and show gender differences^33, 34^. Our present study showed that PEE can lead to foetal over-exposure to maternal glucocorticoids, accompanied with gender differences in the altered hippocampal morphology and expression levels of GAD67 and IGF1. Therefore, we believe that these changes are mainly caused by maternal glucocorticoid over-exposure.

Glucocorticoid participates in the regulation of cell proliferation, differentiation and metabolism, mainly by interacting with a variety of transcription factors (such as C/EBPs) through its corticoid receptors (CRs)^36, 37^. C/EBPs, including C/EBPα, act as one of the important transcription factors that cooperate with glucocorticoid/CR and participate in the rapid regulation of downstream gene expression^38^. C/EBPα has been reported to be able to activate 11β-HSD1 gene transcription^39, 40^, and increased 11β-HSD1 can enhance the intracellular glucocorticoid level by bioactivation of inactive cortisone to active hydrocortisone^41^. Our results showed that glucocorticoid metabolic activation and inactivation co-existed in the male foetal hippocampus with PEE, but the female foetal hippocampus showed typical glucocorticoid metabolic activation (Supplementary Fig. 1). These results suggested that females were more exposed to local active glucocorticoid than males by PEE. High-glucocorticoid levels have been reported to be able to downregulate IGF1 expression by acting on the oestrogen receptor^42, 43^, consistent with the significant inhibition of the expression of IGF1 pathway-related genes in the PEE female foetal hippocampus observed in our study (Supplementary Fig. 1). These observations suggested that the typical glucocorticoid metabolic activation of the PEE female foetal hippocampus may mediate hippocampal developmental damage by inhibiting the IGF1 pathway. Although glucocorticoid metabolic activation of the foetal hippocampus was not obvious in PEE males, GAD67 upregulation can not only promote the conversion of glutamate to GABA and reduce local glutamate excitotoxicity in the hippocampus but also alter the stress onset and sensitivity of the hypothalamus, thereby mediating HPAA hypersensitivity.

Epigenetic modifications exist in normal embryonic and foetal development^44^, and these modifications are sensitive to exogenous environmental factors (e.g., glucocorticoids)^45, 46^. The intrauterine programming of the HPAA mediates the susceptibility to metabolic syndrome in IUGR adult offspring^47^. Studies have demonstrated that there are epigenetic modification abnormalities in diseases related to environmental and genetic interactions^48–51^. Studies have shown that the stable expression of DNMT1 in the hippocampus can cause methylation of the CpG island in the GAD67 promoter to maintain the normal expression of GAD67^52–55^. Furthermore, glucocorticoids can reduce DNMT1 expression in AtT-20 cells in a concentration-dependent manner^56^. In the present study, we confirmed that corticosterone can upregulate GAD67 expression and decrease DNMT1 expression in H19-7 cells, accompanied by a decrease in glutamatergic neurons and increase in GABAergic neurons. What is more, the BSP results further confirmed that the total methylation rate was much lower in the -1019 to -691-bp region of the GAD67 promoter of corticosterone-exposed cells than in that of control cells, while the GR inhibitor RU486 reversed these changes, suggesting that GR mediated these effects of corticosterone. These results implied that the PEE-induced high-glucocorticoid blood level was able to decrease methylation in the -1019 to -691-bp region of the GAD67 promoter by promoting the expression of GR in the foetal hippocampus. The demethylation of the GAD67 promoter and enhanced expression of GAD67 induced by glucocorticoid/GR was maintained until birth and even into adulthood as compensatory responses to the hippocampal injury caused by PEE, mediating the hypersensitivity of the HPAA to CS and increasing the susceptibility to foetus-originated diseases.

**Fig. 8:**
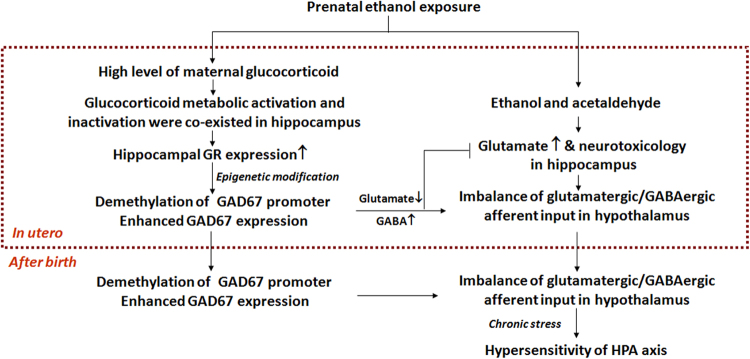
The intrauterine programming mechanism responsible for the hypothalamic–pituitary–adrenal (HPA) axis hypersensitivity in male adult offspring rats with prenatal ethanol exposure. GABA gamma aminobutyric acid, GAD67 glutamic acid decarboxylase 67, GR glucocorticoid receptor

In summary, we proposed that the high-glucocorticoid level mediated the intrauterine programming mechanism responsible for the hypersensitivity of the HPAA in male PEE-induced IUGR offspring (Fig. 8). That is, PEE induced an over-exposure of maternal glucocorticoid in foetal rats and resulted in the demethylation of the GAD67 promoter and upregulation of GAD67 expression through activation of hippocampal GR, thereby promoting the biotransformation of glutamate to GABA in the cytoplasm and vesicles of hippocampal neurons to balance the excitatory/inhibitory neuronal activity and neurotransmitter levels and reducing the excitotoxicity resulting from the ethanol-induced over-release of glutamate in hippocampal neurons (the compensatory effect). However, these intrauterine changes may alleviate the hippocampal excitatory effects on the glutamate-GABA synaptic connections, resulting in weakening negative regulation of the hypothalamus, ultimately leading to an increased excitatory ability of the hypothalamus. Increased expression of GAD67 as a compensatory effect was programmed in utero by epigenetic modifications, which continued until after birth or even into adulthood, and mediated the hypersensitivity of the HPAA to CS in the PEE male offspring.

## Materials and methods

### Animals and treatment

Specific pathogen-free (SPF) Wistar rats (10-weeks-old) (No. 2012-2014, certification number: 42000600002258, license number: SCXK (Hubei)) weighing 180–220 g (females) and 260–300 g (males) were obtained from the Experimental Centre of Hubei Medical Scientific Academy (Wuhan, China). The animal experiments were performed in the Centre for Animal Experiment of Wuhan University (Wuhan, China), which is accredited by the Association for Assessment and Accreditation of Laboratory Animal Care International (AAALAC International). The committee on the Ethics of Animal Experiments of the Wuhan University School of Medicine approved the protocol (permit number: 201719). All animal experimental procedures were performed in accordance with the Guidelines for the Care and Use of Laboratory Animals of the Chinese Animal Welfare Committee.

Animals were housed in metal cages with wire-mesh floors in an air-conditioned room under standard conditions (room temperature: 18–22 °C; humidity: 40%–60%; light cycle: 12-hour light–dark cycle; 10–15 air changes per hour) and allowed free access to rat chow and tap water. All rats were acclimated 1 week before experimentation, and two female rats and one male rat were placed together in a cage for mating overnight. Pregnant rats were randomly divided into either the control group or PEE group. The appearance of sperm in a vaginal smear confirmed mating, and the day of mating was used as gestational day (GD) 0. A schematic illustrating the animal treatment procedure is shown in Supplementary Fig. 4^44^. The pregnant Wistar rats were treated with 4 g/kg per day of ethanol (cat. no. GB678-90, Zhen Xin Co., Ltd., Shanghai, China) by oral gavage from GD9 to GD20, while those of the control group were given an equal volume of saline.

To examine the foetal rats, 16 pregnant rats from each group were euthanized on GD20. Pregnant rats with a litter size of 8–14 pups were deemed to be qualified. The male and female foetuses were quickly removed and weighed. IUGR was diagnosed when the bodyweight of a foetus was two standard deviations lower than the mean bodyweight of the control group^57^. Foetal blood samples were collected to prepare the serum. The foetal hippocampus and hypothalamus were isolated under a dissecting microscope. Eight foetal samples from two pregnant rats were pooled as an independent sample (*n* = 8) and stored at −80 °C for qRT-PCR. The remaining samples were fixed in 2.5% glutaraldehyde (cat. no. GX72-2.5 Yuanmu, Shanghai, China) for transmission electron microscopy analysis, except four foetal brains per group were immediately frozen in liquid nitrogen after they were embedded in Opti-mum cutting temperature compound and then stored at −80 °C for pathological and immunofluorescence analysis.

For the examination of the adult rats, the remaining pregnant rats from each group were allowed to deliver at full term. On postnatal day (PD) 1, the litter size was normalised to 12 pups including six males and six females per litter to assure adequate and standardised nutrition until weaning at postnatal week (PW) 4. After weaning, two male pups were randomly selected from each pregnant rat in both the control and PEE groups (*n* = 8). One of the two male pups was exposed to a 2-week ice-water swimming test (4 to 8 °C for 5 min per day) beginning at PW10. Eleven hours after the last swim, all rats were euthanized at PW12. The serum was obtained and subsequently stored at −80 °C. The hippocampus and hypothalamus were also rapidly collected and stored at −80 °C until further analysis. Four whole brains from each group were randomly selected and processed for pathological and immunofluorescence analysis. Female pups were treated in the same way.

### Serum ACTH and corticosterone detection

The serum ACTH and corticosterone concentrations were measured by the radioimmunoassay kit (cat. no. suer0018, Suer Biological Technology Co. Ltd., Shanghai, China) and the enzyme linked immunosorbent assay (ELISA) assay kit (cat. no. KGE009, R&D Systems Inc., MN, USA), respectively, as previously described^44^. Additionally, the gain rates of serum ACTH and corticosterone were calculated as described below and presented as a percentage (%).

$$\begin{array}{l}{\mathrm A}{\mathrm C}{\mathrm T}{\mathrm H}({\mathrm C}{\mathrm O}{\mathrm R}{\mathrm T}){\it{con}}{\it{.gain rate}}\,{\it{(\% )}}\\ = \frac{{{\mathrm A}{\mathrm C}{\mathrm T}{\mathrm H}({\mathrm C}{\mathrm O}{\mathrm R}{\mathrm T})\,{\mathrm c}{\mathrm o}{\mathrm n}.{\mathrm a}{\mathrm f}{\mathrm t}{\mathrm e}{\mathrm r}\,{\mathrm s}{\mathrm t}{\mathrm r}{\mathrm e}{\mathrm s}{\mathrm s}\, - {\mathrm A}{\mathrm C}{\mathrm T}{\mathrm H}{\it{(}}{\mathrm C}{\mathrm O}{\mathrm R}{\mathrm T}{\it{)}}\,{\mathrm c}{\mathrm o}{\mathrm n}.{\mathrm b}{\mathrm e}{\mathrm f}{\mathrm o}{\mathrm r}{\mathrm e}\,{\mathrm s}{\mathrm t}{\mathrm r}{\mathrm e}{\mathrm s}{\mathrm s}}}{{{\mathrm A}{\mathrm C}{\mathrm T}{\mathrm H}{\it{(}}{\mathrm C}{\mathrm O}{\mathrm R}{\mathrm T}{\it{)}}\,{\mathrm c}{\mathrm o}{\mathrm n}.{\mathrm b}{\mathrm e}{\mathrm f}{\mathrm o}{\mathrm r}{\mathrm e}\,{\mathrm s}{\mathrm t}{\mathrm r}{\mathrm e}{\mathrm s}{\mathrm s}}} \times {\it{100}}{\it{.}}\end{array}$$

### Hippocampal haematoxylin-eosin (HE) staining and transmission electron microscopy (TEM) analysis

Hippocampus tissues stained with HE were processed by standard procedures. The sections (5 μm) were observed and photographed with an Olympus AH-2 light microscope (Olympus, Tokyo, Japan). For TEM analysis, 1-mm^3^ tissue blocks of the hippocampus samples were placed in 3% glutaraldehyde solution with 0.1 M phosphate-buffered solution (PBS). Samples were post-fixed for 1.5 h in 1% osmium tetroxide solution, washed in 0.1 M PBS, dehydrated in graded concentrations of ethanol, and embedded in Epon 618. The epoxy blocks were sliced on an ultramicrotome (LKB-V, LKB, Stockholm, Sweden, 70 nm), stained with uranyl acetate and lead citrate, and examined using a Hitachi H600 transmission electron microscope (Hitachi, Co., Tokyo, Japan). Digital images were computationally acquired.

### Analysis of hypothalamic and hippocampal function-associated gene mRNA expression

Detailed protocols for total RNA extraction, reverse transcription and qRT-PCR were reported in our previous study^32^. The sequences and annealing conditions for the genes are listed in Table 1. The expression levels of the *cellular* genes were calculated using the ∆∆Ct method.

**Table 1:** Rat oligonucleotide primers and reaction conditions used for quantitative real-time PCR

Genes	Forward primers	Reverse primers	Products (bp)	Annealing
*α-CaMKII*	GCATCTGCCGCTTGTTGAA	AGTGTAGCACAGCCTCCAAG	192	58 °C, 20 s
*β-actin*	GTTGCCAATAGTGATGACCT	GGACCTGACAGACTACCTCA	208	54 °C, 20 s
*11β-HSD1*	GGAGCCCATGTGGTATTGA	AGTGCCGGCAATGTAGTGA	105	58 °C, 20 s
*11β-HSD2*	TGGCCAACTTGCCTAGAGAG	TTCAGGAATTGCCCATGC	76	58 °C, 20 s
*AKT1*	ATGAGCGACGTGGCTATTGTGAAG	GAGGCCGTCAGCCACAGTCTGGATG	330	60 °C, 30 s
*AVP*	AAGAGGGCCACATCCGACA	AGGGCAGGTAGTTCTCCTCCTG	160	58 °C, 20 s
*C/EBPα*	CGCAAGAGCCGAGATAAAGC	CCTAGAGATCCAGCGACCCT	270	62 °C, 30 s
*CRH*	AGAACAACAGTGCGGGCTCA	GCTCCGGTTGCAAGAAATTCA	196	60 °C, 30 s
*DNMT1*	GCTAAGGACGATGATGAGACGC	CTTTTTGGGTGACGGCAACTC	447	60 °C, 30 s
*GAD65*	TGCAGCCTTGGGGATCGGAA	CCCCAAGCAGCATCCACATGCA	237	60 °C, 30 s
*GAD67*	CAAGTTCTGGCTGATGTGGA	GCCACCCTGTGTAGCTTTTC	231	60 °C, 30 s
*GR*	CACCCATGACCCTGTCAGTC	AAAGCCTCCCTCTGCTAACC	156	61 °C, 30 s
*IGF1*	GACCAAGGGGCTTTTACTTCAAC	TTTGTAGGCTTCAGCGGAGCAC	148	60 °C, 30 s
*IGF1R*	GTCCTTCGGGATGGTCTA	TGGCCTTGGGATACTACAC	195	60 °C, 30 s
*Mash1*	GAAGATGAGCAAGGTGGAGACG	CGGAGAACCCGCCATAGAGT	169	60 °C, 30 s
*NR1*	TCCTGCTGGTCAGCGACGAC	CCAGCCACACGTACCCAGAG	255	60 °C, 30 s
*NR2A*	GTGATGCCTGTCTGCGGATGG	TAGGAGTGCTGTCGGTTA	169	60 °C, 30 s
*NR2B*	TGGAATGGCATGATCGGTGAG	AGCCACCGCAGAAACAAT	240	60 °C, 30 s
*Pax6*	AAGCAAAATAGCCCAGTATAAACG	TAATGGGTCCTCTCAAACTCTTTC	450	58 °C, 20 s
*PSD95*	TATGTAACGAAGATCATCGAAGGA	GAGAATACGAGGTTGTGATGTCTG	229	58 °C, 20 s
*Reelin*	CAGCAATGGGCTCGTGGTTT	TGTGGGTCTTGTCCTTCTTTT	233	58 °C, 20 s
*Synapsin I*	GTTCTTCGGAATGGGGTCAAA	GAACCATCTGGGCAAACACC	202	60 °C, 30 s
*Tbr2*	CCCCAACAGAGCGAAGAGGT	GGGAAGACAGGTGGGCTCATT	290	58 °C, 20 s
*VGluT2*	TCCACCGGGGTGGCAAAGTT	TGCGATGTATCCGCCCGGAA	128	60 °C, 30 s

*α-CaMKII* Ca^2+^/calmodulin-dependent protein kinase II-α, *11β-HSDs* 11β-hydroxysteroid dehydrogenases, *AKT1* protien kinase B, *AVP* arginine vasopressin, *C/EBPα* CCAAT enhancer-binding protein, *CRH* corticotrophin-releasing hormone, *DNMT1* DNA methyltransferase 1, *GAD* glutamic acid decarboxylase, *GR* glucocorticoid receptor, *IGF1* insulin-like growth factor 1, *IGF1R* insulin-like growth factor receptor 1, *Mash1* mammalian achaete-scutehomolog-1, *NR* *N*-methyl-d-aspartate-subtype glutamate receptor, *Pax6* paired box 6, *PSD95* post-synaptic density 95, *Tbr2* T-box brain protein 2, *VGluT2* vesicular glutamate transporter 2

### Immunohistochemistry analysis of hippocampal GAD67

The immunohistochemical procedures were performed using a streptavidin-peroxidase (SP)-conjugated method according to the manufacturer’s instructions. Paraffin-embedded tissues were cut into 5-μm-thick serial sections and stained with the mouse anti-GAD67 antibody (1:200 dilutions; cat. no. sc-28376, Santa Cruz Biotechnology Inc., Texas, USA). All subfields from each section were examined (*n* = 4). A cytoplasmic brown granule in the neuron cells was marked as positive expression of GAD67. At least five random fields from each section were examined using light microscopy and analysed by HMIAS-2000. Positive content = mean absorbance × positive area.

### Analysis of hippocampal neurotransmitters, glutamate and GABA

The levels of GABA in the hippocampus tissue were measured by the ELISA kit (cat. no. E0900Ge, EIAab science Co. Ltd., Wuhan, China), and the hippocampal glutamate concentration was assayed using a biochemical analysis kit (cat. no. A074, Jianchen Bio-Tek Inc., Nanjing, China), according to the provided protocol.

### Immunofluorescence analysis of hippocampal glutamatergic neurons and GABAergic neurons

The adjacent hippocampal brain sections (10 μm) from the same levels used for the detection of glutamatergic neurons and GABAergic neurons were incubated in 2 N HCl for DNA denaturation (*n* = 3), neutralised with 0.1 M boric buffer (pH 8.5) and then incubated in bovine serum and the primary antibodies: the mouse anti-glutamate (1:2000 dilutions, cat. no. 22523, Immuno Star Inc., WI, USA) or the mouse anti-GAD67 (1:500 dilutions, cat. no.sc-28376, Santa Cruz Biotechnology Inc., Texas, USA) antibody in combination with the rabbit anti-NeuN antibody (1:500 dilutions, cat. no. ab104225, Abcam Inc., MA, USA) for 120 min at 4 °C overnight. The negative control group was incubated with 0.01 M PBS. Then, sections were washed and incubated in the respective secondary antibody: goat anti-rabbit IgG^®^ FITC (1:200 dilutions, cat. no. bs-0295G-FITC, Bioss Biotechnology, Beijing, China) or goat anti-mouse IgG^®^Cy3 (1:200 dilutions, cat. no. 115-166-003, Jackson Immuno Research Laboratories Inc., Baltimore, USA). The stained sections were examined with a Leica fluorescence microscope (Leica, DM5000B; Leica CTR5000; Leica, Germany). The Leica Application Suite Advanced Fluorescence (LAS AF, Leica Microsystems, Germany) software was used for quantification.

### DNA extraction and BSP

Hippocampal GAD67 promoter methylation levels were detected by BSP. Genomic DNA was extracted using a TIANamp Genomic DNA kit (cat. no. DP304, Tiangen Co., Ltd., Beijing, China), Then, the bisulfite conversion of DNA (1 μg) was performed with an EZ DNA Methylation kit (cat. no. D5005, ZYMO RESEARCH, Orange County, CA, USA) according to the manufacturer’s instructions. After the bisulfite treatment, the DNA was diluted to a concentration of 20 ng/μl. The GAD67 primer sequences were designed by MethPrimer software^58^ and were as follows: forward, 5'-TTAGTAYGGGGTTTTTGTGTGTTTG-3'; reverse, 5'-CTATTTCCCTTTCTCTAAACCCTC-3'.Then, 3 μL DNA sample in a 50-μL amplification reaction system, including 5 μL TaKaRa Taq Hot Start (cat. no. DR007A, TaKaRa, Tokyo, Honshu, Japan), was amplified with PCR. Touchdown PCR was used for amplification using the following steps: denaturation at 98 °C for 4 min; an initial heat-start at 98 °C for 4 minutes followed by 20 cycles of 95 °C for 45 s, 66 °C for 45 s, and 72 °C for 45 s and then 20 cycles of 95 °C for 45 s, 56 °C for 45 s, and 72 °C for 30 s; with a final elongation step at 72 °C for 8 min. PCR products were purified using a SanPrep Column PCR Product Purification kit (SK8141, Sangon, Shanghai, China). The purified PCR product then was cloned into a pUCm-T Vector (cat. no. BS433, BBI, Boston, MA, USA). The competent cells were prepared using a One Step Competent Cell Preparation Kit (cat. no. SK9307, Sangon, Shanghai, China) and transformed. Plasmids were extracted with an Endotoxin-Free Plasmid Mini-Preps Kit (cat. no. SK8161, Sangon, Shanghai, China) and sequenced after blue and white screening. The methylation status was analysed using the online BiQ Analyser software (http://biq-analyser.bioinf.mpi-inf.mpg.de/tools/Methylation Diagrams/index.php).

### Rat hippocampus cell line H19-7 culture and treatment

The foetal rat hippocampus cell line H19-7 (ATCC® No. CRL-2526™) grows at 34 °C but differentiates to a neuronal phenotype at 39 °C in DMEM/high-glucose medium supplemented with 10% foetal bovine serum (cat. no. 10100147, Gibco, Carlsbad, CA, USA), 4 mM l-glutamine (cat. no. 25030149, Gibco, Carlsbad, CA, USA), 1.5 g/l sodium bicarbonate (cat. no. 144-55-8, Macklin Biochemical Co., Ltd.), 0.2 mg/ml G418 (cat. no. 108321-42-2, Sigma-Aldrich, Louis, MO, USA) and 0.001 mg/ml puromycin (cat. no. 58-58-2, Sigma-Aldrich, Louis, MO, USA). The cells were transferred to 6-well plates with 3 × 10^5^ cells per well. After incubation of cells at 34 °C and 5% CO_2_ in complete medium for 24 h, the cells were treated with different concentrations of corticosterone (0, 20, 100, 500 ng/ml) (cat. no. C2505, Sigma-Aldrich, Louis, MO, USA) or supplemented with 500 ng/ml corticosterone and different concentrations of mifepristone (0, 5 μM) (cat. no. M8046, Sigma-Aldrich, Louis, MO, USA) and maintained at 39 °C and 5% CO_2_ for 5 days. The complete medium containing the corresponding drugs was replaced every day, and the cells were collected on the 5th day.

### Statistical analysis

SPSS 17 (SPSS Science Inc., Chicago, IL, USA) and Prism 5.0 (GraphPad Software, La Jolla, CA, USA) were used only for data analysis. The quantitative data were expressed as the mean ± S.E.M. Student’s two-tailed *t*-test was performed on one factor of prenatal ethanol treatment. Paired *t*-tests were used to compare the means of the groups without and after CS. The data from cell experiments with different drug concentrations were analysed using one-way ANOVA. A chi-square analysis was performed to test for a difference in the proportions of the categorical variables between groups, such as the methylation rate and the IUGR rate. Statistical significance was defined as *P* < 0.05.

## Electronic supplementary material

Supplymentary Fig. 1

Supplymentary Fig. 2

Supplymentary Fig. 3

Supplymentary Fig. 4

Supplementary figure legends
