# Supplementary figures and images for: Programming for increased expression of hippocampal GAD67 mediated the hypersensitivity of the hypothalamic–pituitary–adrenal axis in male offspring rats with prenatal ethanol exposure
Source: Cell Death Dis. 2018 May 31;9(6):659. doi: 10.1038/s41419-018-0663-1 (PMC5981620; doi:10.1038/s41419-018-0663-1)

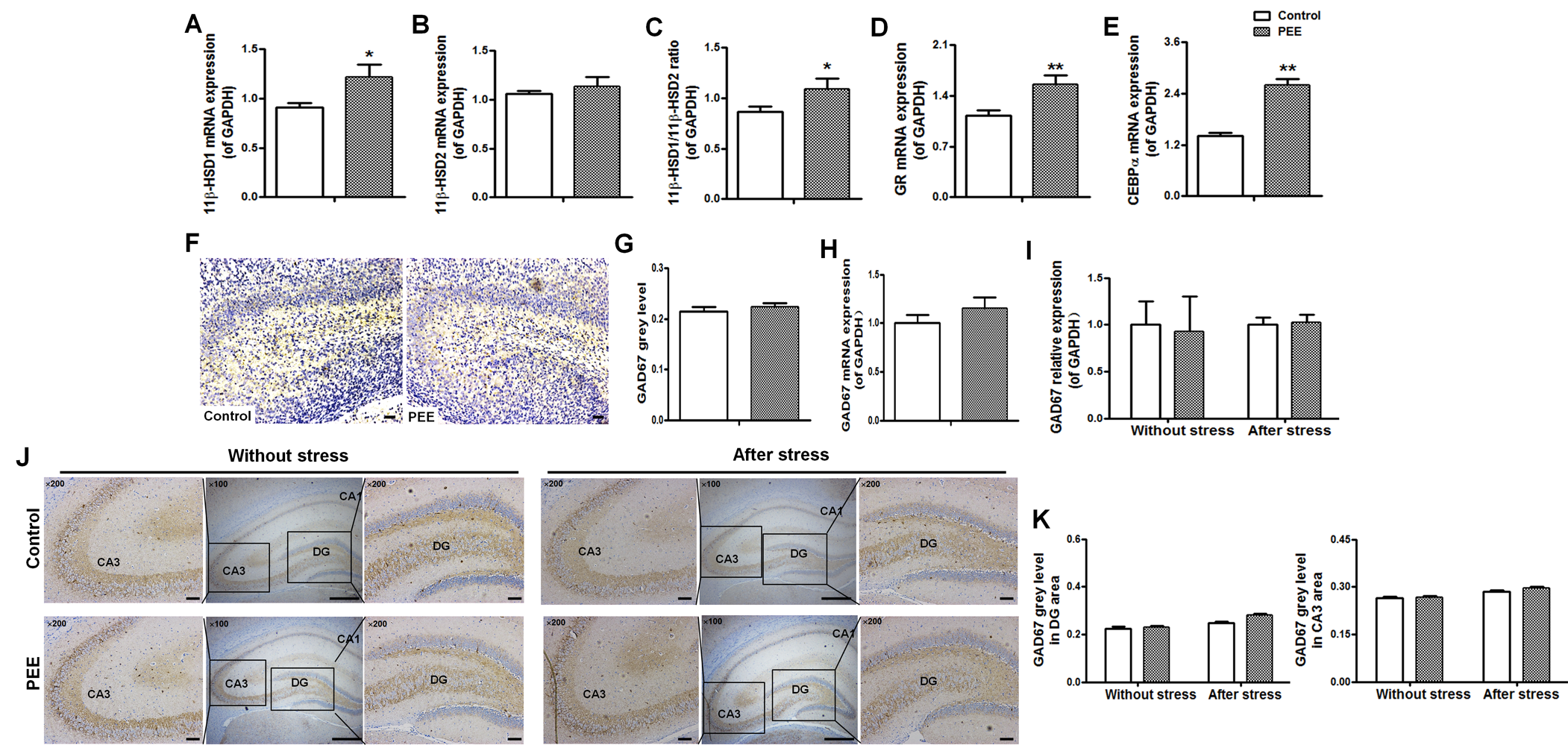

Supplement: Supplementary file 1 — Supplymentary Fig. 1 [file 41419_2018_663_MOESM1_ESM.tif]

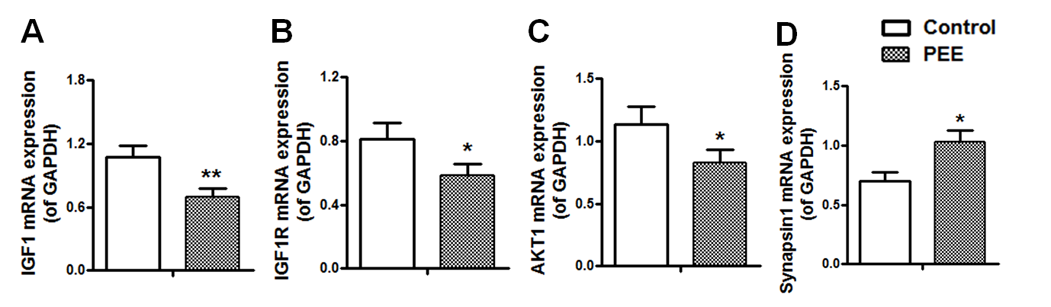

Supplement: Supplementary file 2 — Supplymentary Fig. 2 [file 41419_2018_663_MOESM2_ESM.tif]

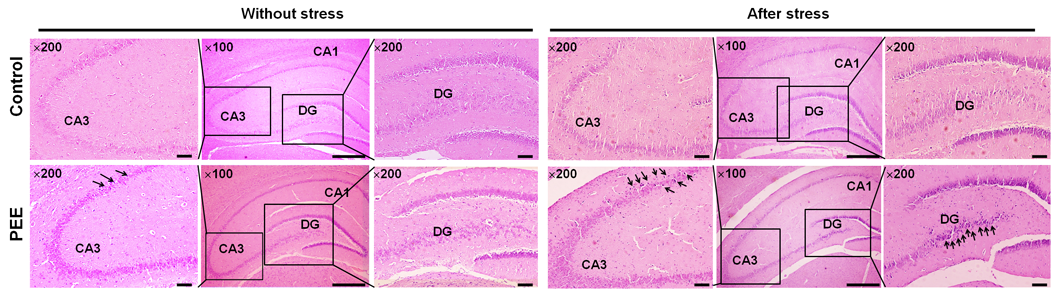

Supplement: Supplementary file 3 — Supplymentary Fig. 3 [file 41419_2018_663_MOESM3_ESM.tif]

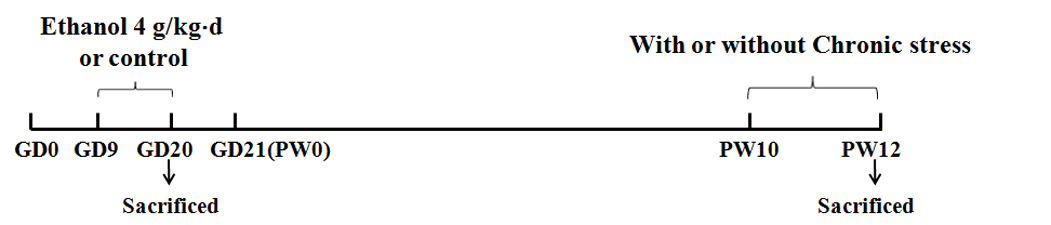

Supplement: Supplementary file 4 — Supplymentary Fig. 4 [file 41419_2018_663_MOESM4_ESM.tif]
