# Supplementary material for: Programming for increased expression of hippocampal GAD67 mediated the hypersensitivity of the hypothalamic–pituitary–adrenal axis in male offspring rats with prenatal ethanol exposure
Source: Cell Death Dis. 2018 May 31;9(6):659. doi: 10.1038/s41419-018-0663-1 (PMC5981620; doi:10.1038/s41419-018-0663-1)
Supplement: Supplementary file 5 — Supplementary figure legends [file 41419_2018_663_MOESM5_ESM.docx]

**Supplementary Figure Legends**

**Supplementary Figure S1** Effects of prenatal ethanol exposure (PEE, 4 g/kg·d) on the expression of components of the hippocampal glucocorticoid metabolic activation system and glutamic acid decarboxylase 67 (GAD67) and the total methylation rate of the GAD67 promoter region (-1019 to -691 bp) in female foetal rats, as well as the expression of hippocampal GAD67 in female adult offspring rats. (A-F) The mRNA expression of components of foetal glucocorticoid metabolic activation system (11β-hydroxysteroid dehydrogenases (11β-HSDs), 11β-HSD1/11β-HSD2 expression ratio, glucocorticoid receptor (GR) and CCAAT enhancer binding protein α (C/EBPα) and GAD67 (n=8 litters). (G, H) Photomicrographs and quantitative analysis of immunohistochemistry for foetal hippocampal GAD67; the brown signal was distributed in the whole hippocampus (n=8 litters). (I) The mRNA expression of GAD67 in female adult offspring rats (n=8 offspring from 8 litters). (J, K) Photomicrographs and quantitative analysis of immunohistochemistry for hippocampal GAD67 in female adult offspring rats; three brain sections from different levels of the hippocampus were selected from each animal and were quantified (n=8 offspring from 8 litters). Mean±S.E.M., ^*^*P*<0.05, ^**^*P*<0.01 *vs*. control.

**Supplementary Figure S2** Effects of prenatal ethanol exposure (PEE, 4 g/kg·d) on hippocampal expression of insulin-like growth factor 1 (IGF1) signal pathway-related genes in female foetal rats. (A-D) Hippocampal expression of IGF1, type 1 insulin-like growth factor receptor (IGF1R), protein kinase B (AKT1) and synapsin 1. Mean±S.E.M., n=8 litters. ^*^*P*<0.05, ^**^*P*<0.01 *vs*. control.

**Supplementary Figure S3** Effects of prenatal ethanol exposure (PEE, 4 g/kg·d) on hippocampal morphology without and after chronic stress in female adult offspring rats. Morphologic changes in the whole hippocampus (HE, ×100), granular cells in the dentate gyrus (DG) areas and pyramidal cells in the cornu ammonis 3 (CA3) areas (HE, ×200). There were only a few neuronal nuclei in the CA3 and DG regions that were dense and darkly stained in the PEE group both without and after CS. n=8 offspring from 8 litters

**Supplementary Figure S4** The schematic procedure of animal treatment. GD, gestational day; PW, postnatal week.
